# Caffeic Acid, One of the Major Phenolic Acids of the Medicinal Plant *Antirhea borbonica*, Reduces Renal Tubulointerstitial Fibrosis

**DOI:** 10.3390/biomedicines9040358

**Published:** 2021-03-30

**Authors:** Bryan Veeren, Matthieu Bringart, Chloe Turpin, Philippe Rondeau, Cynthia Planesse, Imade Ait-Arsa, Fanny Gimié, Claude Marodon, Olivier Meilhac, Marie-Paule Gonthier, Nicolas Diotel, Jean-Loup Bascands

**Affiliations:** 1 Diabète Athérothrombose Thérapies Réunion Océan Indien, INSERM, UMR 1188, Université de La Réunion, 2 Rue Maxime Rivière, 97490 Sainte-Clotilde, France; bryan.veeren@univ-reunion.fr (B.V.); matthieu.bringart@inserm.fr (M.B.); chloe.turpin@univ-reunion.fr (C.T.); philippe.rondeau@univ-reunion.fr (P.R.); cynthia.planesse@univ-reunion.fr (C.P.); olivier.meilhac@inserm.fr (O.M.); marie-paule.gonthier@univ-reunion.fr (M.-P.G.); nicolas.diotel@univ-reunion.fr (N.D.); 2 Groupe d’Intérêt Public, Cyclotron Réunion Océan Indien, 2 Rue Maxime Rivière, 97490 Réunion, France; i.aitarsa@cyroi.fr (I.A.-A.); f.gimie@cyroi.fr (F.G.); 3 Aplamedon Réunion, CYROI-Parc Technor—2, Rue Maxime Rivière, 97490 Réunion, France; claude.marodon@aplamedom.org

**Keywords:** *Antirhea borbonica*, kidney fibrosis, polyphenols, caffeic acid, antioxidant enzymes, DESI-imaging

## Abstract

The renal fibrotic process is characterized by a chronic inflammatory state and oxidative stress. *Antirhea borbonica* (*A. borbonica*) is a French medicinal plant found in Reunion Island and known for its antioxidant and anti-inflammatory activities mostly related to its high polyphenols content. We investigated whether oral administration of polyphenol-rich extract from *A. borbonica* could exert in vivo a curative anti-renal fibrosis effect. To this aim, three days after unilateral ureteral obstruction (UUO), mice were daily orally treated either with a non-toxic dose of polyphenol-rich extract from *A. borbonica* or with caffeic acid (CA) for 5 days. The polyphenol-rich extract from *A. borbonica*, as well as CA, the predominant phenolic acid of this medicinal plant, exerted a nephroprotective effect through the reduction in the three phases of the fibrotic process: (i) macrophage infiltration, (ii) myofibroblast appearance and (iii) extracellular matrix accumulation. These effects were associated with the mRNA down-regulation of *Tgf-β*, *Tnf-α*, *Mcp1* and *NfkB*, as well as the upregulation of *Nrf2*. Importantly, we observed an increased antioxidant enzyme activity for GPX and Cu/ZnSOD. Last but not least, desorption electrospray ionization-high resolution/mass spectrometry (DESI-HR/MS) imaging allowed us to visualize, for the first time, CA in the kidney tissue. The present study demonstrates that polyphenol-rich extract from *A. borbonica* significantly improves, in a curative way, renal tubulointerstitial fibrosis progression in the UUO mouse model.

## 1. Introduction

A common deleterious consequence of most chronic kidney diseases (CKD) is the interstitial accumulation of extracellular matrix leading to tubulointerstitial fibrosis, which is closely correlated with the loss of renal function [1,2,3,4]. The process of renal fibrosis can be seen as an ongoing wound-healing process maintained by a chronic inflammatory reaction [5,6]. Halting renal fibrosis progression appears as a relevant therapeutic strategy to at least delay CKD progression. Although significant progress in the understanding of the molecular mechanisms occurring during fibrosis has been made [6], specific antifibrotic drugs and/or treatments are still clearly lacking. To the best of our knowledge the only drugs used in clinic to slow down the progression of CKD are angiotensin-converting enzyme inhibitors and angiotensin type I receptor blockers [7,8,9]. Diabetic kidney disease remains the main cause of CKD, leading to end-stage kidney disease in both type 1 and type 2 diabetes [10]. Recently, sodium–glucose linked transporter-2 (SGLT2) inhibitors appeared to be the most promising nephroprotective drugs in diabetic kidney disease [11,12]; however, an antifibrotic effect has only been evidenced in animal studies [13]. There is thus a need and ample space to develop new therapeutic approaches targeting tubulointerstitial fibrosis in the kidney for combatting these pathological processes [14].

Chronic infiltration of immune cells in renal tissue, as well as chronic hypoxia and oxidative stress, are clearly involved in the initiation and progression of the chronic fibrosis process. At present, targeting chronic inflammation and oxidation seems to be a reasonable option to slow down renal fibrosis progression. Indeed, a number of experimental studies have demonstrated the efficiency of targeting inflammation and oxidative stress [14,15,16,17]. However, translation to the clinic is either missing or has been, most of the time, disappointing due to non-desirable side effects [14,15,16,17]. In addition, due to the chronicity of renal diseases one might wonder if a long-term specific anti-inflammatory treatment would promote susceptibility to infection. Taken together, it seems that targeting a single factor or pathway is not sufficient to efficiently prevent renal tubulointerstitial fibrosis. Therefore, it is understandable that a growing number of studies have investigated the health benefit of traditional medicine, also called natural/herbal medicine, including medicinal plants, which can be considered as a “multidrug” therapy. However, regarding the use of herbal medicine in the prevention and treatment of CKD, and more precisely tubulointerstitial fibrosis, very few studies have been carried out mainly due to the dramatic consequences associated with the ingestion of Chinese herbal medicine containing aristolochic acid [18,19]. It is thus very important to perform rigorous preclinical investigations to assess the presence of nephrotoxic molecules, as well as the beneficial and/or deleterious/toxic effects of these plants/molecules, particularly in the context of kidney disease.

In Reunion Island, since 2012, 27 medicinal plants have been registered at the French pharmacopeia [20] (https://ansm.sante.fr/, accessed on 30 March 2021). Most of them are known for their antioxidant and anti-inflammatory activities related mostly to their polyphenols content. However, although a number of studies (mostly in vitro studies) have reported various potential therapeutic effects such as antihypertensive [21], antioxidant, anti-inflammatory [22], antiviral [23], antiplasmodial and anti-Chikungunya effects [24], as well as an inhibitory effect on Dengue and Zika virus infection [25], the in vivo validation phase for further medical uses of these effects on preclinical models is missing.

*Antirhea borbonica* (*A. borbonica*) leaves are peculiarly interesting, as they are widely used in traditional medicine for treating, among others, diabetes, urinary tract infection, diarrhea, hemorrhage, rheumatism and also kidney stones [26,27]. Our laboratory has shown that *A. borbonica* exhibited strong antioxidant and anti-inflammatory effects, in vitro, on preadipocytes, cerebral endothelial cells and red blood cells [22,28,29]. Those antioxidant and anti-inflammatory biological effects were mainly associated with the capacity of polyphenols to down-regulate on one hand key molecular targets such as IL6, MCP-1 and NF-kB, and on the other hand increase superoxide dismutase (SOD) as well as the redox-sensitive translational factor Nrf2. In addition, the in vitro studies showed that predominant polyphenols such as quercetin, chlorogenic and caffeic acids were able to reduce free radicals through DPPH and AAPH radical-scavenging tests [22,28,29].

More recent data obtained in vivo also highlight a preventive protective effect of *A. borbonica* aqueous extract in a zebrafish diet-induced overweight model in displaying cerebral oxidative stress and blood–brain barrier leakage [30], as well as in a mouse stroke model [31]. Registration at the French pharmacopeia supposes that the medicinal plants are devoid of toxic effects in humans. Most of the time, this statement is based on ethnobotany investigations, mainly oral information/folk knowledge, claiming that the consumption of the plant is free from toxicities and side effects. However, regarding *A. borbonica,* to the best of our knowledge, no in vivo preclinical study investigating a putative nephroprotective effect has been reported. We have recently reported a detailed (qualitative and quantitative) phenolic profile as well as the antioxidant activity of aqueous and organic extracts of *A. borbonica* and determined the LC_50_ of both extracts on a zebrafish embryos model [32] according to the OECD (Organisation for Economic Co-operation and Develpoment) guidelines, allowing us to safely investigate in vivo the effect of *A. borbonica* in a kidney disease context.

Because of the potential therapeutic interest of *A. borbonica* against renal tubulointerstitial fibrosis, but also because we have previously reported [32] that the main polyphenols of *A. borbonica* was caffeic acid (CA) and since it is now well admitted that the absorption of CA derivatives results in free CA as secondary metabolites [33], the aims of our study were (i) to look for the presence of nephrotoxic molecules, (ii) to evaluate the renal antifibrotic effect of *A. borbonica* as well as CA in the in vivo unilateral ureteral obstruction (UUO) mouse model and (iii) to investigate the presence of putative specific antifibrotic molecules from *A. borbonica* at the renal tissue level.

## 2. Materials and Methods

### 2.1. Chemicals and Reagents

Folin–Ciocalteu reagent, sodium carbonate, sodium nitrite, aluminum chloride, 2,2-Diphenyl-1-picrylhydrazyl (DPPH) and caffeic acid (CA) were purchased from Sigma Aldrich (St. Louis, MO, USA). Solvents such as acetone, acetonitrile and methanol were purchased from Carlo Erba (Peypin, France).

### 2.2. Plant Material

*Antirhea borbonica* J.F Gmelin (*A. borbonica*) powder prepared from the dried leaves was obtained from the APLAMEDOM institute (Association pour les Plantes Aromatiques et Médicinales de la Réunion) and registered under the following code: DéTROI.002/2018, stating the date of collection and the GPS coordinates (21°05′44.9” S, 55°39′06.6” E), altitude: 770 m. The pharmacist and director of APLAMEDOM performed the botanical identification of *A. borbonica*. *A. borbonica* powder was stored at −20 °C until polyphenol extraction.

### 2.3. Nephrotoxic Compounds Identification and Quantification of Polyphenols by UPLC-UV-ESI-MS/MS

Polyphenolic extract from *A. borbonica* was prepared by dissolving 1 g of crushed leaves in 25 mL of an aqueous acetonic solution (70%, *v/v*). After incubation at 4 °C for 90 min, the mixture was centrifuged at 3500× *g* rpm at 4 °C for 20 min and polyphenol-rich supernatant was collected and stored at −80 °C until analysis. Identification of polyphenols was carried out by ultra-high-performance liquid chromatography (UHPLC) coupled with diode array detection and a HESI-Orbitrap mass spectrometer (Q Exactive Plus, Thermo Fisher). A 10 µL sample volume was injected using an UHPLC system equipped with a Thermo Fisher Ultimate 3000 series WPS-3000 RS autosampler and then separated on a PFP column (2.6 μm, 100 mm × 2.1 mm, Phenomenex, Torrance, CA, USA). The column was eluted with a gradient mixture of 0.1% formic acid in water (A) and 0.1% formic acid in acetonitrile (B) at the flow rate of 0.450 mL/min, with 5% B at 0.00 to 0.1 min, 35% B at 0.1 to 7.1 min, 95% B at 7.2 to 7.9 min and 5% B at 8.0 to 10 min. The column temperature was held at 30 °C and the detection wavelengths were set to 280 and 310 nm.

For mass spectrometer conditions, a Heated Electrospray Ionization source II (HESI II) was used. Nitrogen was used as drying gas. The mass spectrometric conditions were optimized as follows: spray voltage = 2.8 kV, capillary temperature = 350 °C, sheath gas flow rate = 60 units, aux gas flow rate = 20 units and S lens RF level = 50. Mass spectra were registered in full scan mode from *m*/*z* 100 to 1500 in negative ion mode at a resolving power of 70,000 FWHM at *m*/*z* 400. The automatic gain control (AGC) was set at 1e6. The MS/MS spectra were obtained by applying a relatively higher energy collisional dissociation (HCD) energy of 25%.

Identification of the compounds of interest was based on their retention time, exact mass, elemental composition, MS fragmentation pattern and comparisons with available standards and the advanced mass spectral database, *m*/*z* Cloud (https://www.mzcloud.org, accessed on 3 February 2021). The search for nephrotoxic compounds was carried out based on their exact mass in the MS spectrum (Extract Ions Chromatograms (XICs)). Data were acquired by XCalibur 4.2 software (Thermo Fisher Scientific Inc., Waltham, MA, USA) and processed with compound discoverer 2.1, and Skyline 20.1 software (MacCoss Lab., Seattle, WA, USA) was used to confront raw files with our “in house” database.

### 2.4. Desorption Electrospray Ionization-High Resolution/Mass Spectrometry (DESI-HR/MS) Imaging

The 2D automated Omni Spray Kidney tissues were flash-frozen in nitrogen and stored at −80 °C before DESI-HR/MS imaging. For DESI-MS imaging and histology, 12 μm thickness kidney sections were collected and mounted on SuperFrost™ Plus glass slides.

The 2D automated Omni Spray ion source from Prosolia Inc. (Indianapolis, IN, USA) coupled to a Q-Exactive™ Plus mass spectrometer (Thermo Fisher Scientific, Bremen, Germany) was used to perform the mass spectrometry imaging experiment. A solution of methanol with 0.1% formic acid (HPLC-MS grade, Carlo Erba) was used as the extraction and ionization spray solvent, delivered by a syringe pump at a flow rate of 5 μL/min. All imaging experiments were carried out with the following experimental conditions including source parameters: 2.8 kV capillary voltage, 250 °C capillary temperature, 60% S-lens RF level and 86 psi nitrogen nebulizing gas pressure, and including geometrical parameters: ∼1 mm spray tip-to-surface distance and a spray incident angle of 60°. Mass spectra were registered in full scan mode with the mass spectrometer operating in negative mode. Survey full scan mass spectra were acquired in the 50 to 500 *m*/*z* range at resolving power 70,000 (at *m*/*z* 400) with an automatic gain control (AGC) target of 3^6^ and maximum injection time of 200 ms. DESI-HR/MS imaging of tissues was performed in start point-constant velocity scan mode, with a scan rate of 185.2 µm/s and a spatial resolution of 100 μm.

Mass spectra were acquired using XCalibur 4.2 software (Thermo Fisher Scientific Inc.).The XCalibur mass spectral files (.raw) were converted to mZML then to imzML [34]. MSIQuant software [35] was used to generate the selected ion images.

Periodic acid-Schiff (PAS) staining was performed on the same tissue sections after DESI-MSI to visualize tissue structure.

### 2.5. Animal Model: Unilateral Ureteral Obstruction (UUO)—Biodistribution and Pharmacokinetic Studies

All reported experiments were performed at the GIP-CYROI technological platform’s animal facility (A974001), conducted in accordance with NIH guidelines for the care and use of laboratory animals, and were approved by the French authorities (APAFIS#7347-2016100314466830v5, approved on 4 September 2017). C57BL/6J mice (male, 6 weeks old) were purchased from Janvier, (Le Genest Saint Isle, France) and housed in a pathogen-free, temperature-controlled environment with a 12–12 h light/dark photocycle. Animals had free access to food and tap water, to avoid dehydration-related hypovolemia. All mice were fed with a normal diet.

The unilateral ureteral ligation was performed as previously described [36]. Briefly, under oxygen–isoflurane anesthesia and through a longitudinal, left abdominal incision, the ureter was exposed and ligated with a 6/0 nylon thread at the uretero–pelvic junction. In sham operations, the ureter was exposed but not ligated and repositioned. To reduce pain Buprenorphine (0.01 mg/kg) (Buprecare centravet, Maison-Alfort, France) was injected i.p before surgery and 12 h later. Except for the preventive experiment where *A. borbonica* polyphenol-rich extract was administered by gavage 1 day before UUO and then every day for 5 days, the treatment with *A. borbonica* polyphenol extract (25 mg/kg) or CA (25 mg/kg) was initiated 3 days after UUO and continued for 5 days. *A. borbonica* polyphenol extract (25 mg/kg) or CA (25 mg/kg) were resuspended in distilled water just before gavage. The control group received only the vehicle (distilled water). At the end of the different protocols, mice were sacrificed and the kidneys were removed, and a transverse section was fixed in Carnoy’s solution for 24 h and subsequently embedded in paraffin for immuno-histological analysis. Several pieces of renal cortex were snap-frozen in liquid nitrogen and stored at −80 °C for mRNA, enzyme activities and MS/MS analysis.

For biodistribution study, one day before sacrifice animals were placed in metabolic cages to collect urine overnight. CA and its metabolites were measured by UPLC-MS/MS in liver and kidney tissues and urine.

For pharmacokinetics study, the animals were submitted to UUO and 3 days later they were fasted overnight and then treated with a single dose of CA (25 mg/kg) or vehicle (*n* = 3/group). Blood samples (90 µL) were collected at time 0 (before treatment), 30 min, 60 min, 180 min and 360 min post-treatment. CA concentrations were measured by UPLC-HESI-Q-orbitrap (Q Exactive Plus).

### 2.6. Determination of Phenolic and Flavonoid Contents—Measurement of the Total Antioxidant Capacity of Polyphenol-Rich Plant Extract Administered Orally

The total phenolic acid content in *A. borbonica* extract was determined by using the Folin–Ciocalteu assay [37] and expressed as mg gallic acid equivalent (GAE) per 100 g dry plant powder. The total flavonoid content was determined by using the aluminum chloride (AlCl_3_) colorimetric assay [38] and expressed as mg quercetin equivalent (QE) per 100 g dry plant powder.

The total antioxidant capacity of *A. borbonica* extract was assessed through the 2,2-Diphenyl-1-picrylhydrazyl (DPPH) radical scavenging assay using vitamin C positive control. The absorbance (Abs) was read at 517 nm (FLUOstar Optima, Bmg Labtech). The percentage of free radical-quenching activity of DPPH was calculated from the following formula:

Antioxidant capacity (%) = [(Abs_vehicle_ − Abs_sample_)/Abs_vehicle_] × 100

### 2.7. Immunohistochemistry and Histological Analysis

Kidneys were fixed in Carnoy’s solution, dehydrated and embedded in paraffin. From kidney sections, routine histology and immunohistological staining and analysis were performed as previously described [39]. Three to four µm thickness sections were cut and used for routine staining (hematoxylin–eosin and Sirius red staining) and immunohistochemistry. The extent of Sirius red staining on the kidney section was scored from 0 to 4+ as follows: 0: no staining; 0.5: <10%; 1: 10–25%; 2: 25–50%; 3: 50–75%; and 4: >75%. For immunohistochemistry, mouse renal tissue was first de-waxed in toluene and rehydrated through a series of graded ethanol washes before incubation with 3% hydrogen peroxide for 10 min at room temperature to block endogenous peroxidase activity. Specific primary antibodies were incubated (1 h at room temperature) on mouse kidney sections for the detection of macrophages with anti-mouse F4/80 (RM2900; Caltag laboratories Inc., Burlingame, CA, USA; dilution 1/250); rabbit anti-alpha smooth muscle actin antibodies (ab5694-abcam; dilution 1/250). The sections were subsequently stained with the Dako Envision system (K4000 (primary antibodies from mouse) and K4002 (primary antibodies from rabbit)) according to manufacturer’s instruction. Sections were finally counterstained with hematoxylin. Negative controls for the immunohistochemical procedures included substitution of the primary antibody with nonimmune sera at a similar immunoglobin concentration. Sections were scanned using a NanoZoomer S60 (Hamamatsu) and image analysis was realized in a blind fashion using Image J software (https://imagej.nih.gov/ij/, accessed on 30 March 2021).

### 2.8. RT-qPCR

Total RNA was isolated from mouse kidneys with TRIzol^TM^ reagent (Invitrogen). One microgram of total RNA was reverse transcribed with random hexamer primers and Superscript II reverse transcriptase (Applied Biosystems). The quantitative real-time polymerase chain reaction was performed in a 96 well plate using SYBR green^TM^ master mix (Eurogentec). Analysis of GAPDH was performed to normalize gene expression and the relative mRNA fold changes between groups were calculated using the 2^−^^ΔΔ^^Ct^ method. Primer sequences are listed in Table 1.

### 2.9. Protein Isolation from Kidney Tissue, Antioxidant Activities (Mn-SOD, Cu/Zn-SOD, GPX, CAT) and Protein Carbonylation

For enzymatic activities determination, protein isolation from kidney tissue was performed as follow: between 10 to 30 mg of kidney tissues previously collected and stored at −80 °C were homogenized with a TissueLyser II (Qiagen) in 150 µL of Tris buffer (Tris (25 mM), EDTA (1 mM), pH 7.4). After centrifugation (5000× *g*/min, for 10 min), the supernatant was used for protein quantification and enzymatic assays. The total protein level of lysate was quantified by the bicinchoninic acid assay (BCA).

SOD activity was assayed by monitoring the rate of acetylated cytochrome c reduction by superoxide radicals generated by the xanthine/xanthine oxidase system. Measurements were performed in a reagent buffer (xanthine oxidase, xanthine (0.5 mM), cytochrome c (0.2 mM), KH_2_PO_4_ (50 mM), EDTA (2 mM), pH 7.8) at 25 °C. The specific MnSOD activities were determined in the same conditions after incubation of samples with NaCN (1 mM) which inhibits Cu/ZnSOD activities. Assays were monitored by spectrophotometry at 560 nm. SOD activities were calculated using a calibration standard curve of SOD (up to 6 unit/mg). Total SOD, MnSOD and resulting Cu/ZnSOD activities were expressed as international catalytic units per mg of proteins.

The total activity of glutathione peroxidase (GPX) was determined with cumene hydroperoxide as substrate. The rate of glutathione oxidized by cumene hydroperoxide (6.5 mM) was evaluated by the decrease in NADPH (0.12 mM in Tris buffer) at 340 nm in the presence of NaCN (10 mM), reduced glutathione (0.25 mM) and glutathione reductase (1 U/mL) in Tris buffer (50 mM, pH 8). GPX activity was expressed as international units per gram of proteins.

The catalase (CAT) activity assay was carried out on 40 µg of protein lysate in 25 mM Tris–HCl (pH 7.5). Blanks were measured at 240 nm just before adding 80 µL of H_2_O_2_ (10 mM final) to start the reaction. Catalase activity was determined by measuring the absorbance at 240 nm and was calculated using a calibration standard curve of an increasing amount of catalase between 12.5 and 125 unit/mL. Catalase activity was expressed as international catalytic units per mg of proteins.

The protein carbonylation was determined as described previously [40] by the carbonyl ELISA assay based on the recognition of protein-bound DNPH in carbonylated proteins with an anti-DNP antibody.

### 2.10. Statistics

Individual data are presented as dot plots next to the average for the group. Comparison between two groups of values was achieved by using a two-tailed unpaired Welch’s t-test. For statistical comparisons involving more than two experimental groups, analysis of variance (ANOVA) followed by Dunnett’s test was used. Statistical analyses and the determination of the area under the curve (AUC _(0-360)_) were performed with Graph-Pad Prism 6.3 (GraphPad Software, Inc., San Diego, CA, USA). Data are mean ± SD. A *p*-value < 0.05 was considered statistically significant.

## 3. Results

### 3.1. Detection and Identification of Nephrotoxic Components

In the present study, based on their exact mass in MS spectrum, we focused on the detection and identification of known nephrotoxic molecules (Table S1) in the polyphenol-rich plant extract from *A. borbonica*. None of these molecules were found in the *A. borbonica* extract. This reassuring result prompts us to investigate, in vivo, the putative nephroprotective effect of a non-toxic concentration of polyphenol-rich extract from *A. borbonica.*

### 3.2. Phenolic/Flavonoid Contents and Total Antioxidant Activity of Orally Administered Polyphenol-Rich Extract from A. borbonica.

**Figure 1:**
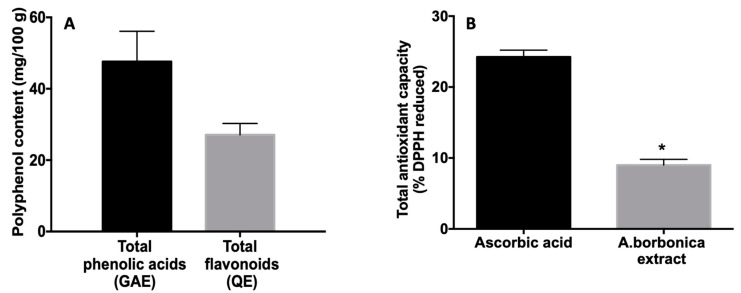
Total phenolic acid and flavonoid contents (**A**) and antioxidant capacity (**B**), from *A. borbonica* extract administered by gavage. (**A**) Total phenolic contents were determined by using the Folin–Ciocalteu colorimetric assay and total flavonoid contents were determined by using the aluminum chloride colorimetric assay. The results are expressed as mg gallic acid equivalent (GAE)/100 g and as mg quercetin equivalent (QE)/100 g plant dried powder. (**B**) Total antioxidant capacity was measured by DPPH assay. Positive control ascorbic acid was used at the same phenolic acid concentration (47 mg GAE) as that provided by the *A. borbonica* extract. The results are expressed as % of reduced DPPH. Data are means ± SD of three independent experiments. * *p* < 0.05 vs. ascorbic acid.

Based on our previous study [32], the dose of 25 mg/kg was chosen for mice gavage and corresponded to the lowest dose (1.4 g/L) tested on the zebrafish (embryos/larvae) model, showing no toxicological effect. As shown in Figure 1A, the quantity of polyphenol administered by gavage is not negligible since we found total contents of phenolic acids and flavonoids of 47.6 ± 8.5 mg GAE/100 g dried powder and of 27.1 ± 3.2 mg QE/100 g dried powder, respectively. In addition, *A. borbonica* extract exhibited an antioxidant capacity accounting for 37% of the positive control ascorbic acid (Figure 1B).

### 3.3. Preventive Experiment: Effects of Polyphenol-Rich Extract from A. borbonica on Body and Kidney Weight and Diuresis in a UUO Model

In order to ascertain that the 5 days of treatment with *A. borbonica* (25 mg/kg) did not result in unexpected adverse effects, we investigated the body weight before and after the treatments, and the diuresis and the left kidney weight at the end of the experiments. As shown in Table 2, neither the UUO treatment nor the UUO + *A. borbonica* treatment led to a significant change in animal body weight, kidney weight and diuresis.

### 3.4. Preventive Effect of Polyphenol-Rich Extract from A. borbonica in UUO-Induced Tubulointerstitial Fibrosis

**Figure 2:**
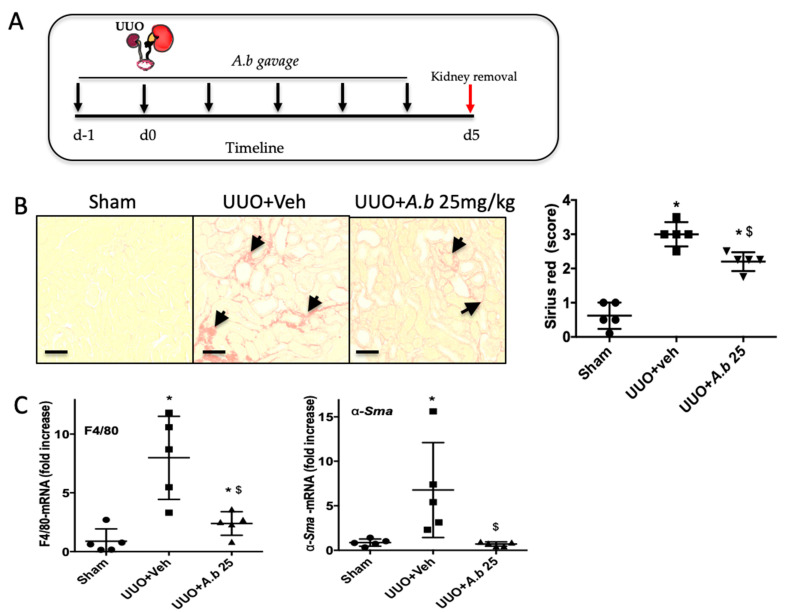
Preventive effect of *A. borbonica* (*A. b*) 25 mg/kg on renal tubulointerstitial fibrosis induced by unilateral ureteral obstruction (UUO) in mice. (**A**) Experiment design: black arrows indicate daily gavage with 25 mg/kg *A. b.* (**B**) Renal tubulointerstitial fibrosis highlighted with the Sirius red staining, arrows indicate interstitial fibrosis. (**C**) qPCR analyses of mRNA–macrophage infiltration (F4/80) and myofibroblasts (*α-Sma*); *n* = 5 per group. * *p* < 0.05 compared to Sham; $ *p* < 0.05 compared to UUO + Veh. Scale bar =100 μm.

To determine the possible nephroprotective effect of polyphenol-rich extract from *A. borbonica,* we administrated it by gavage one day before the UUO and allowed the mice to survive for 5 days with a daily *A. borbonica* gavage. The sham group received the vehicle (distilled water). At the end of the experimental procedure (day 5), we assessed interstitial collagen deposition by the analysis of Sirius red-stained renal sections (Figure 2A). In this “proof of concept” study, identification of a nephroprotective effect leads us to pursue the investigation, otherwise we do not go any further.

Pretreatment with polyphenol-rich extract from *A. borbonica* (25 mg/kg) for 5 days significantly decreased tubulointerstitial collagen accumulation as shown by the Sirius red staining (Figure 2B). Because interstitial collagen accumulation is strongly associated with an increase in macrophage infiltration (F4/80) in the kidney and the appearance of myofibroblasts (α-SMA), we examined the mRNA expression of these two markers. Whereas UUO increased the gene expression of *F4/80* and *α-SMA*, both markers were significantly decreased in the group treated with polyphenol-rich extract from *A. borbonica* (Figure 2C).

This very first experiment performed on a reduced number of animals and the rapid evaluation of the three phases of the fibrotic process (inflammation, myofibroblasts appearance and interstitial collagens accumulation) allowed us to make the decision to continue the evaluation of the polyphenol-rich extract from *A. borbonica*.

### 3.5. Curative Effect of Polyphenol-Rich Extract from A. borbonica in UUO-Induced Tubulointerstitial Fibrosis.

**Figure 3:**
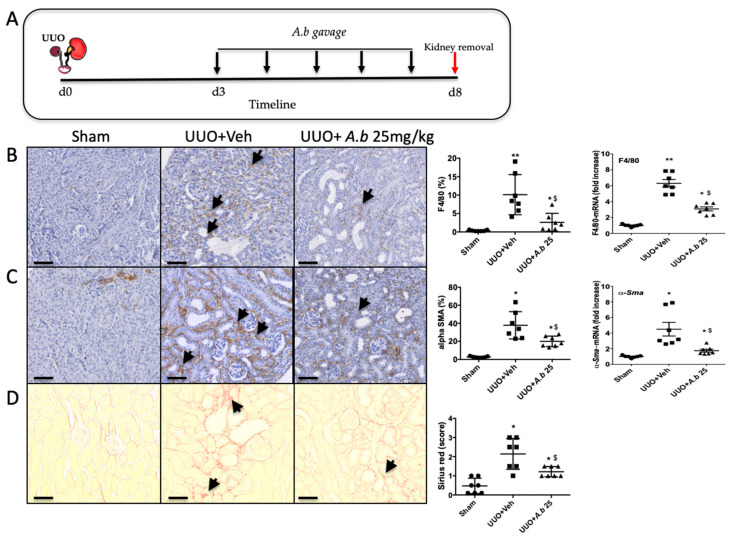
Curative effect of *A. borbonica* (*A. b*) 25 mg/kg on renal tubulointerstitial fibrosis induced by unilateral ureteral obstruction (UUO) in mice. (**A**) Experiment design: the black arrows indicate daily gavage with *A. b.* (**B**) Macrophage infiltration revealed by F4/80 immunostaining, arrows indicate macrophages. (**C**) Accumulation of myofibroblasts revealed by alpha SMA staining, arrows indicate myofibroblasts. (**D**) Renal tubulointerstitial fibrosis shown by Sirius red staining, arrows indicate interstitial fibrosis. Sham-operated group (Sham), unilateral ureteral obstruction group receiving vehicle (UUO + Veh) and unilateral ureteral obstruction group receiving polyphenol-rich extract from *A. b* (UUO + *A. b* 25 mg/kg). For each staining, quantitative results are shown on the right part of the figure (scatter dot plot), as well as renal mRNA expression of *F4/80* and *α-Sma*; *n* = 7 per group. * *p* < 0.05, ** *p* < 0.01, compared to Sham; $ *p* < 0.05 compared to UUO + Veh. Scale bar = 100 μm.

To truly evaluate the nephroprotective effect of polyphenol-rich extract from *A. borbonica,* we administrated the solution, by gavage, 3 days after surgery for 5 days (Figure 3A). *A. borbonica* extract significantly reduced macrophage infiltration and myofibroblast appearance, assessed by F4/80 (Figure 3B) and α−SMA (Figure 3C) immunostaining, respectively, and confirmed by qPCR analysis. Moreover, *A. borbonica* extract decreased extracellular matrix accumulation assessed by Sirius red staining (Figure 3D).

**Figure 4:**
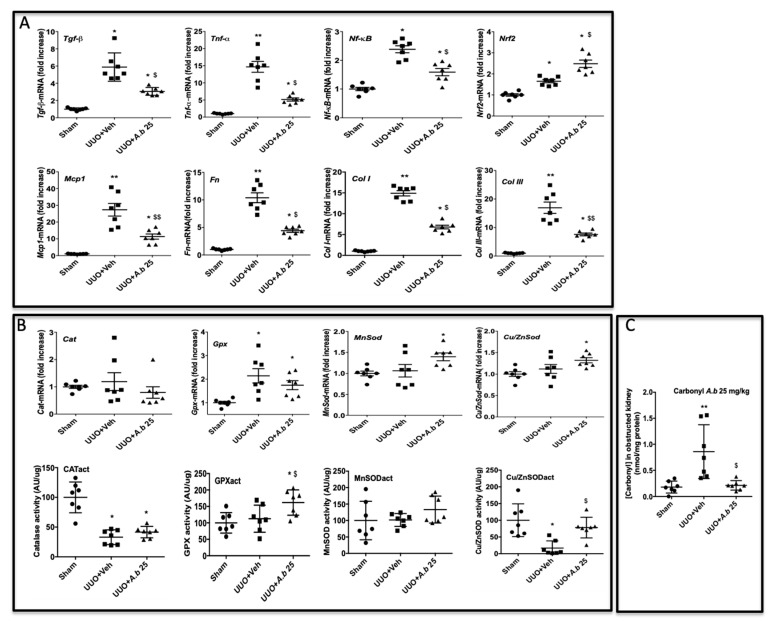
*A. borbonica* (*A. b*) extract restores normal inflammatory and oxidative stress states induced by unilateral ureteral obstruction (UUO). (**A**) Expression of genes involved in inflammatory and fibrotic responses in obstructed kidney, (**B**) antioxidant enzyme expression and activity (act) and (**C**) protein carbonyl concentration in obstructed kidney. *n* = 7 per group. * *p* < 0.05, ** *p* < 0.01, compared to Sham; ^$^ *p* < 0.05, ^$$^ *p* < 0.01 compared to UUO + Veh.

To better understand the molecular mechanisms by which *A. borbonica* could reduce renal fibrosis, we analyzed the expression of genes encoding key proteins known to be involved in UUO. Shown in Figure 4A is the UUO-induced overexpression of the mRNA of profibrotic cytokines (*TGF-β*, *TNF-α*) and *NF-κB*, a nuclear factor known to control cytokines production. We also observed the overexpression of *Nrf2*, a redox-sensitive nuclear transcription factor involved in the regulation of antioxidant enzyme genes expression. The mRNA *Mcp-1* encoding for the chemokine MCP-1 (also known as CCL2) which is involved in monocyte/macrophage recruitment was overexpressed by UUO. As expected, UUO induced the expression of fibronectin, type I and III interstitial collagens’ mRNA. Oral administration of *A. borbonica* extract blunted significantly the UUO-induced expression of most of these genes. Interestingly, *A. borbonica* extract induced a significant increase in the mRNA expression of *Nrf2*, suggesting the stimulation of the antioxidant defense system.

These results prompt us to investigate the gene expression and enzyme activities of catalase (CAT), glutathione peroxidase (GPX) and Mn- and Cu/Zn-superoxide dismutase (SOD). As shown in Figure 4B, gene expression did not change for *Cat. Gpx* was increased by UUO whereas *Mn*- and *Cu/Zn-SOD* expression was not modified when compared to the sham group. *A. borbonica* extract treatment significantly increased *Mn*- and *Cu/Zn-SOD* mRNA expression compared to the sham group, but this increase was not significant when compared to the UUO untreated group. Interestingly, we found that, when compared to the UUO group, *A. borbonica* extract treatment was associated with a significant increase in both GPX and Cu/Zn-SOD enzyme activities. More precisely, UUO did not change the GPX enzyme activity but significantly decreased Cu/Zn-SOD activity, and *A. borbonica* extract administration significantly increased GPX and brought back to control values Cu/Zn-SOD activities.

Protein carbonylation is considered as a major hallmark of oxidative damage. The disturbance of redox homeostasis in the UUO model could explain the significant increase in protein carbonyl level in obstructed kidneys, as shown in Figure 4C. Interestingly, this increase was totally suppressed by *A. borbonica* extract treatment (Figure 4C).

### 3.6. Biodistribution of Caffeic Acid and Its Metabolite 24 Hours after Administration of A. borbonica Polyphenol-Rich Extract (25 mg/kg) in Mice Kidney, Liver and Urine

In the next step, we aimed at evaluating the biodistribution of the main polyphenols of *A. borbonica* and caffeic acid derivatives by using mass spectrometry, in order to identify metabolites that may explain the previously observed biological effects. As shown in Table 3, the caffeic acid (CA) was detected as a secondary metabolite in the UUO animals treated with *A. borbonica* polyphenol extract, and was significantly higher in obstructed kidneys when compared to sham and vehicle groups (0.144 ± 0.013 vs. 0.006 ± 0.006 µM, respectively, *** *p* < 0.001). Similarly, significantly elevated concentrations of the methylated form of CA, namely ferulic acid (FA), were detected in the *A. borbonica*-treated UUO mice group (1.119 ± 0.132 vs. 0.076 ± 0.060/0.064 ± 0.011 µM, respectively, *** *p* < 0.001). No metabolite difference was noticed between each group for liver samples. Nonetheless, the highest concentrations of CA and FA were found in urine (96.599 ± 19.704 vs. 38.002 ± 5.021 µM, respectively), indicating excretion, at least in part, by the kidneys. As expected, *A. borbonica* polyphenols were detected in the liver. Caffeic acid and its secondary metabolite FA reach the systemic circulation to be delivered to organs. These findings prompt us to investigate the effect of the CA molecule in this kidney fibrosis model.

### 3.7. Caffeic Acid (CA) Administration Mimics A. borbonica Extract’s Nephroprotective Effects

The presence of CA in mice obstructed kidney (Table 3) and the fact that CA and derivatives were the most abundant polyphenolic compounds of *A. borbonica* [32] suggests that CA could mimic *A. borbonica* extract’s nephroprotective effects.

**Figure 5:**
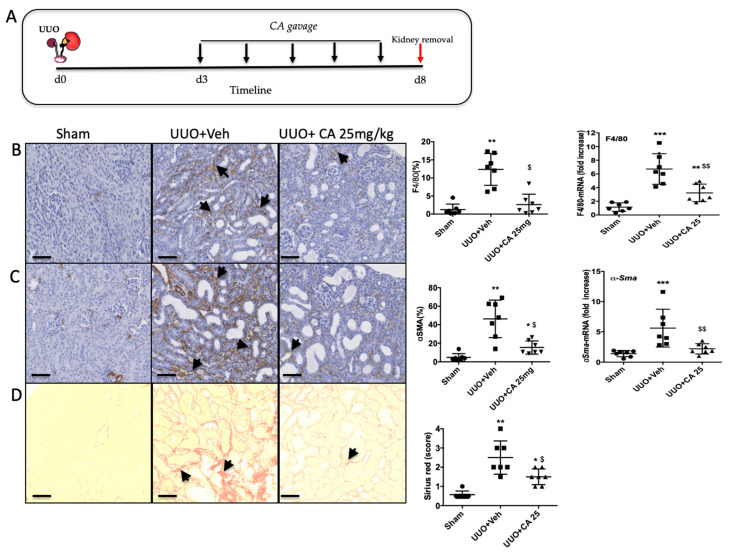
Curative effect of caffeic acid (CA) 25 mg/kg on renal tubulointerstitial fibrosis induced by UUO in mice. (**A**) Experiment design, black arrows indicate daily gavage with CA. (**B**) Macrophage infiltration F4/80, arrows indicate macrophages. (**C**) Accumulation of myofibroblasts alpha SMA, arrows indicate myofibroblasts. (**D**) Tubulointerstitial fibrosis (Sirius red), arrows indicate interstitial fibrosis. Sham operated group (Sham), unilateral ureteral obstruction group receiving vehicle (UUO + Veh) and unilateral ureteral obstruction group receiving caffeic acid (UUO + CA 25 mg/kg). For each staining quantitative results are shown on the right part of the figure (scatter dot plot), as well as renal mRNA expression of F4/80 and α-Sma; *n* = 7 per group. * *p* < 0.05, ** *p* < 0.01, *** *p* < 0.001, compared to Sham; ^$^ *p* < 0.05, ^$$^ *p* < 0.01 compared to UUO + Veh. Scale bar = 100 μm.

We thus investigated if we could reproduce the observed nephroprotective effects of *A. borbonica* by daily oral administration of CA from day 3 after UUO for 5 days (Figure 5A).

**Figure 6:**
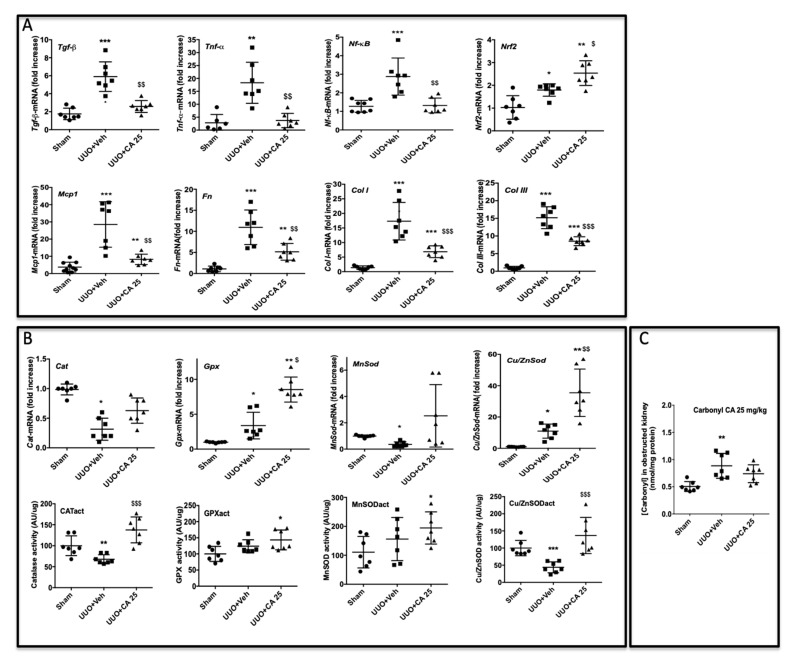
Caffeic acid (CA) 25 mg/kg restores normal inflammatory and oxidative stress parameters induced by UUO in mice. (**A**) Expression of genes involved in inflammatory and fibrotic responses in obstructed kidney, (**B**) antioxidant enzymes gene expression and activities and (**C**) the protein carbonyl concentration in obstructed kidney. *n* = 7 per group. * *p* < 0.05, ** *p* < 0.01, *** *p* < 0.001, compared to Sham; ^$^ *p* < 0.05, ^$$^ *p* < 0.01, ^$$$^ *p* < 0.001, compared to UUO + Veh.

Immunohistological analysis showed a significant protective effect of CA on macrophage infiltration (Figure 5B), accumulation of myofibroblasts (Figure 5C) and renal tubulointerstitial fibrosis (Figure 5D). The overexpression of the mRNA levels of genes involved in the inflammatory and fibrotic responses in obstructed kidneys was downregulated by CA, except for the transcription factor *Nrf2* (Figure 6A), as previously described above for *A. borbonica*. We also observed that CA significantly increased CAT and Cu/ZnSOD enzyme activities (Figure 6B). However, the stimulation of the activity of these antioxidant enzymes by CA resulted in a slight decrease in carbonyl level which did not reach statistical significance (Figure 6C).

Taken together these data strongly suggest that the phenolic compound CA found in *A. borbonica*. could actively participate in the observed nephroprotective effects in the UUO model.

### 3.8. Plasma and Kidney Pharmacokinetic of Caffeic Acid

To evidence that CA participates in the nephroprotective effects of *A. borbonica* extract, we decided to undertake plasma and kidney pharmacokinetics of CA in order to optimize the visualization protocol of the CA presence in the obstructed kidneys by using DESI-HR/MS imaging. To this end, three days after UUO, mice were administrated by oral route a unique dose of CA (25 mg/kg) and then were sacrificed at 30, 60, 180 and 360 min. Caffeic acid (CA) and ferulic acid (FA) concentrations in both the plasma and kidney were assessed by using UPLC-HESI-Q-orbitrap (Q-Exactive™ Plus).

**Figure 7:**
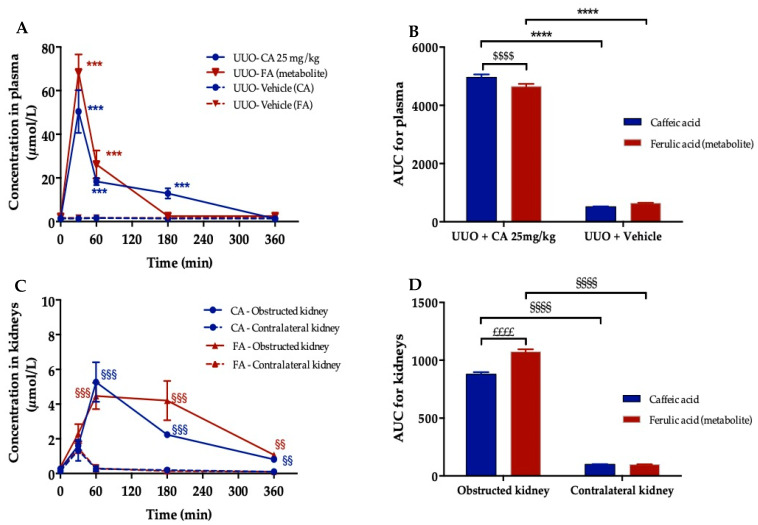
Pharmacokinetic study of caffeic acid (CA) and ferulic acid (FA) after oral administration of CA (25 mg/kg) in 3 days-UUO mice. (**A**) Plasma concentration–time profiles of CA and its circulation metabolite, ferulic acid (FA), in UUO mice. (**B**) Corresponding area under the curves (AUC) of CA and FA in plasma. (**C**) Concentration–time profiles of CA and FA in obstructed and contralateral kidneys. (**D**) Corresponding area under the curves (AUC) of CA and FA in obstructed and contralateral kidneys. Concentrations were determined using UPLC-HESI-Q-orbitrap (Q-Exactive™ Plus). *** *p* < 0.001, **** *p* < 0.0001, compared to UUO + Vehicle; ^$$$$^ *p* < 0.0001, compared to caffeic acid; ^§§^ *p* < 0.01, ^§§^^§^ *p* < 0.001, ^§§^^§§^ *p* < 0.0001, compared to contralateral kidney; ^££££^ *p* < 0.0001, compared to caffeic acid. Values are means ± SD; *n* = 3 animals/time.

As shown in Figure 7A and Table 4, CA exhibited a significant peak plasma concentration of 50.5 ± 9.8 µM (C_max_) within 30 min (*t*_max_) suggesting its rapid absorption. However, due to hepatic first-pass metabolism, a limited bioavailability was noticed (0.4%). Indeed, a significant ferulic acid (FA) peak plasma concentration (68.3 ± 8.3 µM) appeared at the same *t*_max_ as CA. Comparison of the area under the curve (AUC) for plasma showed a significantly higher AUC _(0-360)_ for CA than FA for UUO-CA treated mice (4964 ± 97.3 vs. 4648 ± 91.1, respectively), suggesting that CA stays longer in plasma than FA (Figure 7B). Notably, no plasma CA and FA peak was registered for the vehicle group. In the kidneys (Figure 7C,D), one hour after CA administration, CA and FA concentrations were significantly higher (x5) in the obstructed kidneys compared to the contralateral kidneys (5.3 ± 1.1 and 4.5 ± 0.8 µM vs. 0.3 ± 0.1 and 0.4 ± 0.1 µM, respectively). Interestingly, a significantly high AUC _(0-360)_ was calculated for CA and FA in the obstructed kidneys compared to the contralateral kidneys (881 ± 17.2 and 1073 ± 21 vs. 100 ± 2 and 98.5 ± 11, respectively) indicating their accumulation in the obstructed kidney (Figure 7D). Taking these data into account we set up a DESI-HR/MS experiment to visualize the spatial distribution of CA in the obstructed kidney 1 h after CA gavage.

### 3.9. Visualization of Orally Administered Caffeic Acid (CA) in the Obstructed Kidney

**Figure 8:**
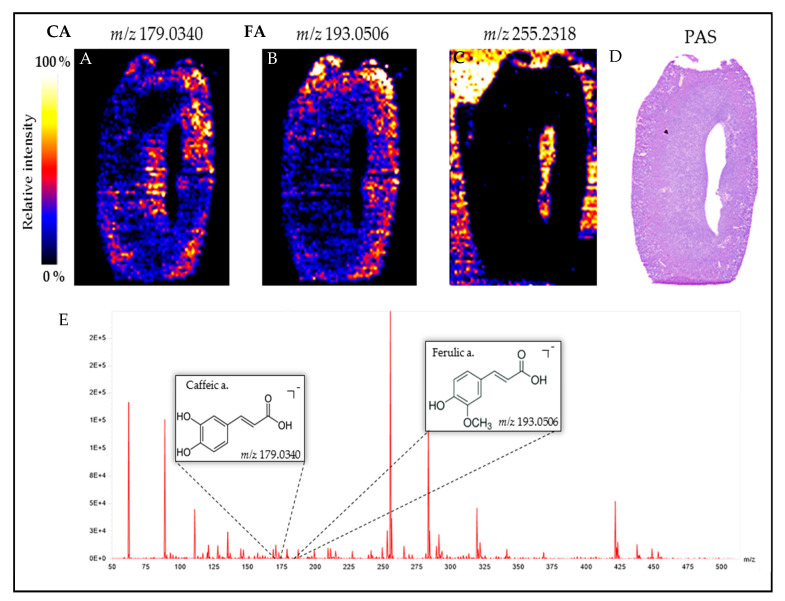
Visualization of caffeic acid (CA) and its circulating metabolite, ferulic acid (FA), 1 h post oral administration of CA 25 mg/kg in the obstructed kidney tissue of mice. Desorption electrospray ionization-high resolution/mass spectrometry (DESI-HR/MS) Imaging of (**A**) caffeic acid (*m/z* 179.0340), (**B**) ferulic acid (*m/z* 193.0506) and (**C**) *m/z* 255.2318 used to show the negative fingerprint of tissue and the corresponding Periodic Acid Schiff (PAS) staining (**D**). (**E**) DESI-HR/MS mass spectrum ranging from *m/z* 50 to 500. The Synapt Blue-Red-Yellow color scale was used.

The spatial distribution of CA (*m*/*z* 179.0340) and FA (*m*/*z* 193.0506) in mice obstructed kidney is shown in Figure 8A,B respectively. When compared to PAS staining (Figure 8D) we observed the presence of CA in the renal cortex where the dilated tubules are found, and thus where tubulointerstitial fibrosis will appear and progress. Caffeic acid is also present in renal papilla, suggesting CA urinary elimination. Taken together our data highlight that CA exerts an antifibrotic effect in the UUO mouse model.

## 4. Discussion

Medicinal plants are usually perceived as safe medication. However, plants can also contain many toxic substances that may be a risk to the kidneys [41,42]. Here, we first looked for the presence of known nephrotoxic molecules and showed that no toxic molecule, at least associated with Chinese herbal nephrotoxicity [43], was present in *A. borbonica.* These results made us confident to investigate *in vivo* the putative nephroprotective effect of *A. borbonica*.

Although rarely found in humans, the complete obstruction of the ureter in the UUO model mimics, in an accelerated manner, the different stages leading to renal tubulointerstitial fibrosis [44]. It includes inflammation associated with macrophage infiltration and the up-regulation of pro-fibrotic molecules, as well as the appearance and the accumulation of myofibroblasts, which are the main cell type responsible for the secretion of extracellular matrix proteins [44]. Because of the highly reproducible fibrotic response this UUO model is now well recognized to test the antifibrotic potency of candidate molecules [45].

In the present study, we provide evidence that the oral administration of polyphenol-rich extract from *A. borbonica* significantly attenuates, in vivo, renal interstitial fibrosis. We showed that this nephroprotective effect goes through reduction in the three phases of the fibrosis process (macrophage infiltration, myofibroblast appearance and extracellular matrix accumulation). An increasing number of studies using the UUO model have reported similar effects, mainly observed in a preventive way, from polyphenol-rich extracts of various origins such as *Elsholtzia ciliate* [46] and *Nigella sativa* [47], but also from polyphenol molecules such as curcumin from *Curcuma longa* [48], icariin, an active flavonoid from the *Epinedium* genus [49], resveratrol [50], quercetin, a flavonoid present in vegetables and fruits [51], and epigallocatechin-3-gallate, a green tea polyphenol [52]. In line with these studies, our results show, for the first time, that either in a preventive or curative treatment *A. borbonica* significantly attenuates macrophage infiltration. This anti-inflammatory effect leads to a strong decrease in myofibroblast appearance in the tubulointerstitial space and consequently to a reduced tubulointerstitial fibrosis, as evidenced by the down-regulation of *Fn* mRNA, as well as *Col I* and *III* at the mRNA and protein levels. As expected, this was associated with the mRNA down-regulation of pro-inflammatory and pro-fibrotic cytokines (*Tgf-β*, *Tnf-α*), a chemokine (*Mcp1*), and a transcription factor (*Nf-κB*).

As polyphenols are known to exert antioxidant effects [53], we studied the expression of *Nrf2*, which is a major transcription factor involved in the regulation of antioxidant enzymes [54,55]. We found that UUO induced an increase in *Nrf2* gene expression. This up-regulation was expected since UUO is known to induce oxidative stress [56], which in turn stimulates Nrf2 gene and protein expression [57]. In addition, we observed that polyphenol-rich extract from *A. borbonica* further stimulated *Nrf2* gene expression. It suggests that polyphenols from *A. borbonica* have the capacity to stimulate *Nrf2*. This result was consistent with the, now well admitted, effect of polyphenols such as Nrf2 activator [58]. To further get insights into the nephroprotective mechanism of polyphenol-rich extract from *A. borbonica*, we measured antioxidant enzymes known to be up-regulated by Nrf2. Whereas polyphenol-rich extract from *A. borbonica* was without significant effect on the mRNA expression of *Cat*, *Gpx* and *Sod* when compared to the UUO-untreated animals, we observed a significant increase in the enzyme activities of GPX and Cu/ZnSOD. Although most of the studies using the UUO model to investigate the renal effects of polyphenols have focused on the protein expression of antioxidant enzymes [49,59], our data are consistent with the few works that investigated the antioxidant enzyme activity, showing that the renal enzyme activities of CAT and total-SOD are significantly reduced in the obstructed kidney [47,60]. The decrease in these main antioxidant activities in the UUO model are generally associated with an increase in reactive oxygen species which can lead to protein damage and the formation of oxidized compounds such as carbonylated proteins [61]. Whereas polyphenol-rich extract from *A. borbonica* had no effect on CAT and Mn-SOD enzyme activities, our data show a significant increase in GPX and Cu/ZnSOD enzyme activities. These results specify more clearly the mechanism of action of *A. borbonica.* In addition, the beneficial effect of *A. borbonica* on both enzymes’ activities seems to be strong enough to protect proteins from the oxidative damage induced by UUO.

We have previously reported [32] that CA and derivatives were the most abundant polyphenolic compounds of *A. borbonica*. In order to explore the mechanism of action of polyphenol-rich extract from *A. borbonica*, we investigated if we could reproduce the observed curative nephroprotective effects of *A. borbonica* extract by oral administration of CA. It is now well known that CA exhibits antioxidant and anti-inflammatory properties [62], and that administration of caffeamide derivatives have shown antifibrotic effects in renal ischemia reperfusion [63], as well as in the UUO model [64]. However, to the best of our knowledge, the oral administration of the CA molecule has never been studied in the UUO model. Since CA is the main phenolic acid found in *A. borbonica* [32], to properly investigate if CA participates in the antioxidant and anti-fibrotic effects observed with the polyphenol-rich extract from *A. borbonica*, we had to study the effect of oral administration of CA in the representative UUO renal tubulointerstitial fibrosis model. Our results clearly show that oral administration for 5 days of CA (25 mg/kg), 3 days after UUO, significantly decreased macrophage infiltration, myofibroblast appearance and extracellular matrix accumulation. This was associated with the down-regulation of pro-inflammatory and pro-fibrotic cytokines, as well as a significant increase in *Nrf2* mRNA expression and CAT and Cu/ZnSOD enzyme activities. This increase in *Nrf2* is consistent with in vitro data showing that CA [65] and CA derivatives [66] exert their protective effect via the Nrf2 pathway. The results obtained with CA administration on antioxidant enzyme activities are slightly different from the effect observed with *A. borbonica* polyphenol-rich extract. Indeed, when compared to the UUO + Veh group, we observed a significant increase in CAT activity, and the increase in GPX activity was not significantly different. In addition, we did not observe a significant effect on carbonylated proteins. In fact, the observed effect on antioxidant enzyme activity with the polyphenol-rich extract from *A. borbonica* may result from the combination of the different polyphenols’ effects. Thus, these discrepancies could be related to the administered CA dose, which is much higher than the CA concentration in the polyphenol-rich extract from *A. borbonica.*

To provide more evidence that polyphenols can exert their biological effects at the kidney level, we used the DESI imaging technique to visualize and provide evidence that CA, given by oral route, is rapidly present in the kidney, strongly suggesting bioavailability of CA in the kidney. Our data are consistent with the biodistribution study of Omar et al. [67] which used [3-^14^C]trans-caffeic acid. However, we observed a low bioavailability (F = 0.4%) probably due to an important metabolic activity in the intestine and liver prior to reaching the main blood stream. The presence of the CA metabolite ferulic acid, produced by the action of catechol-O-methyl transferase, confirms this hypothesis as previously reported [68,69]. DESI-HR/MS imaging allowed us to visualize, for the first time, CA and FA in the renal cortex. Regardless of the quantity of CA that reached the kidney tissue, our data strongly suggests that CA is involved in the nephroprotective effect of the polyphenol-rich extract from *A. borbonica.*

## 5. Conclusions

The present study demonstrates, for the first time, that both polyphenol-rich extract from *A. borbonica* and CA, which is the predominant polyphenol, significantly improves, in a curative way, renal tubulointerstitial fibrosis in the UUO mouse model, especially via their antioxidant and anti-inflammatory properties. Further studies will be necessary to validate this antifibrotic effect on chronic models of renal disease, such as diabetic nephropathy. Notably, we cannot rule out that part of the observed nephroprotective effects of *A. borbonica* could be mediated by other polyphenols present in the plant extract, such as quercetin and kaempferol. Indeed, further investigations will be necessary to determine the individual and synergistic effects between the main polyphenols found in *A. borbonica*, to better understand this nephroprotective effect before considering clinical trials.

## Figures and Tables

**Table 1:** Primers used for reverse transcribed-quantitative polymerase chain reaction (RT-qPCR). Transforming growth Figure 4. nuclear factor erythroid 2-related factor 2 (*Nrf2*); monocyte chemotactic protein (*Mcp1*); fibronectin (*Fn*); collagen type I and III (*Col I, Col III*); alpha smooth muscle actin (*α-Sma*); catalase (*Cat*); glutathione peroxidase (*Gpx*); manganese-dependent superoxide dismutase (*MnSOD*); copper/zinc superoxide dismutase (*Cu/ZnSOD*).

Mouse Gene	Sequence	
*Tgf-β*	Forward Reverse	CCTGAGTGGCTGTCTTTTGA CGTGGAGTTTGTTATCTTTGCTG
*Tnf-α*	Forward Reverse	TCCCAGGTTCTCTTCAAGGGA ACAAGGTACAACCCATCGGC
*Nf-κB*	Forward Reverse	GTGATGGGCCTTCACACACA CATTTGAACACTGCTTTGACT
*F4/80*	Forward Reverse	ACCACAATACCTACATGCACC AAGCAGGCGAGGAAAAGATAG
*Nrf2*	Forward Reverse	TCCCATTTGTAGATGACCATGAG CCATGTCCTGCTCTATGCTG
*Mcp1*	Forward Reverse	GCAGTTAACGCCCCACTCA CCAGCCTACTCATTGGGATCA
*Fn*	Forward Reverse	CTTTGGCAGTGGTCATTTCAG ATTCTCCCTTTCCATTCCCG
*Col I*	Forward Reverse	CATAAAGGGTCATCGTGGCT TTGAGTCCGTCTTTGCCAG
*Col III*	Forward Reverse	GAAGTCTCTGAAGCTGATGGG TTGCCTTGCGTGTTTGATATTC
*α-Sma*	Forward Reverse	GTGAAGAGGAAGACAGCACAG GCCCATTCCAACCATTACTCC
*Gapdh*	Forward Reverse	CTTTGTCAAGCTCATTTCCTGG TCTTGCTCAGTGTCCTTGC
*Cat*	Forward Reverse	CCTCCTCGTTCAGGATGTGGTT CGAGGGTCACGAACTGTGTCAG
*Gpx*	Forward Reverse	TGCTCATTGAGAATGTCGCGTCTC AGGCATTCCGCAGGAAGGTAAAGA
*MnSod*	Forward Reverse	ATGTTGTGTCGGGCGGCG AGGTAGTAAGCGTGCTCCCACACG
*Cu/ZnSod*	Forward Reverse	GCAGGGAACCATCCACTT TACAACCTCTGGACCCGT

**Table 2:** Effect of *A. borbonica* (*A. b*) extract on body weight, left kidney weight and diuresis. Values are means ± SD; *n* = 7 animals/group.

Group	*n*	Body Weight (g)	Left Kidney Weight (g)	Diuresis/24 h (mL)
Sham	7	22.02 ± 2.64	0.134 ± 0.01	1.06 ± 0.44
UUO + veh	7	21.58 ± 1.54	0.136 ± 0.01	1.26 ± 0.6
UUO + *A. b* 25 mg/kg	7	20.49 ± 1.20	0.122 ± 0.008	1.06 ± 0.6

**Table 3:** Distribution of caffeic acid (CA) and its methylated metabolite, ferulic acid (FA), in obstructed kidney, liver and urine after ingestion of 25 mg/kg of *A. borbonica* (*A. b*). The concentrations were measured by UPLC-HESI-Q-Orbitrap (Q-Exactive™ Plus) and expressed in (µM). ** *p* < 0.01, *** *p* < 0.001, compared to UUO + Vehicle. Values are means ± SD; *n* = 7 animals/group.

Concentration (mM)	Sham	UUO + Vehicle	UUO + *A. b* 25 mg/kg
**Kidney obstructed**			
Caffeic acid	0.006 ± 0.005	0.006 ± 0.006	0.144 ± 0.013 ***
Ferulic acid	0.076 ± 0.060	0.064± 0.011	1.119 ± 0132 ***
**Liver**			
Caffeic acid	0.026 ± 0.006	0.031 ± 0.003	0.037 ± 0.001
Ferulic acid	0.391 ± 0.074	0.388 ± 0.104	0.426 ± 0.099
**Urine**			
Caffeic acid	24.912 ± 6.401	21.161 ± 6.856	96.599 ± 19.704 ***
Ferulic acid	10.376 ± 3.505	12.551 ± 3.307	38.002 ± 5.021 **

**Table 4:** Pharmacokinetic parameters at the dose 25 mg/kg of caffeic acid (CA) orally administered in UUO mice. AUC represents the calculated area under the curve between 0 and 360 min. C_max_ is the maximum concentration achieved. *t*_max_ is the required time to achieve C_max_. Bioavailability (F) is the fraction of the administered dose that is available to the systemic circulation. Values are means; *n* = 3 animals/time.

Parameter	Oral Administration (Gavage)
	Caffeic Acid
AUC _(0–360)_	4964
C_max_ (μM)	50.5
*t*_max_ (min)	30
F (%)	0.4

## Supplementary Materials

The following are available online at https://www.mdpi.com/article/10.3390/biomedicines9040358/s1, Table S1: The known nephrotoxic molecules not detected by UPLC-HESI-Q-Orbitrap (Q-Exactive^TM^ Plus) in *A. borbonica* extract.
