# Supplementary material for: Caffeic Acid, One of the Major Phenolic Acids of the Medicinal Plant Antirhea borbonica, Reduces Renal Tubulointerstitial Fibrosis
Source: Biomedicines. 2021 Mar 30;9(4):358. doi: 10.3390/biomedicines9040358 (PMC8065974; doi:10.3390/biomedicines9040358)
Supplement: Supplementary file 1 [file biomedicines-09-00358-s001.pdf]

Table S1. The known nephrotoxic molecules **not detected** by UPLC-HESI-Q-Orbitrap (Q-Exactive™ Plus) in *A. borbonica* extract.

Aristolochic Acid	Anthraquinone	Alkaloids	Miscellaneous
Aristolochic acid I	Alizarin	Calycanthine	Podophyllotoxin
Aristolochic acid II	Damnacanthal	Tetrandrine	Alisol A 24 acetate
	Morindone	Dauricine	Bakuchiol (terpene)
	Rubiadin	Brucine	Geniposide (iridoid)
	Rubiadin 1-methyl ether	Strychnine	Esculentoside A (saponine) triterpene
	Galiosin	Veratrine	
	Munjistin	Aconitine	
	Pseudopurpurin	Pyrrolizidine	
	Alizarin-2-O-Glucoside		
	Alizarin-O-β-glucuronide		
	Alizarin-1-O-sulfation		
	Xanthopurpurin		
	Purpurin-1-O-β-glucuronide		
	2-Methyl-1,3,6-hydroxy-9,10-Anthraquinone-O-β-D-glucoside		
	Nordamnacanthal		
	6-Hydroxyrubiadin		
	Lucidin		
	Lucidin ethyl ether		
	Lucidin w-methyl ether		
